# Microsurgical varicocelectomy for intratesticular varicocele with a history of orchiopexy for undescended testis

**DOI:** 10.1002/iju5.12498

**Published:** 2022-06-16

**Authors:** Teppei Takeshima, Kimitsugu Usui, Shinnosuke Kuroda, Takashi Kawahara, Mitsuru Komeya, Jun‐ichi Teranishi, Hiroji Uemura, Yasushi Yumura

**Affiliations:** ^1^ Department of Urology, Reproduction Center Yokohama City University Medical Center Yokohama Kanagawa Japan; ^2^ Department of Urology and Renal Transplantation Yokohama City University Medical Center Yokohama Kanagawa Japan

**Keywords:** cryptorchidism, intratesticular varicocele, orchiopexy, undescended testis, varicocelectomy

## Abstract

**Introduction:**

We report a case series of six patients with a chief complaint of infertility who underwent microsurgical varicocelectomy for intratesticular varicocele (ITV) after undergoing orchiopexy for undescended testis in their childhood.

**Case presentation:**

The patients' median age was 34 years. The median age at which orchiopexy was performed was 4.5 years. All the cases of ITV were accompanied by extratesticular varicocele (ETV). All the patients underwent a microsurgical subinguinal varicocelectomy. At 3 months after operation, the ITV and ETV had disappeared in all the cases. Sperm density and motility increased in five and four patients, respectively.

**Conclusion:**

ITV occurs at a relatively high frequency after orchiopexy for undescended testis. Disorder of the valvular function of the testicular vein in peeling the testicular vessels was thought to be a primary cause and is often found on the left side. Doppler ultrasonography of the testis is considered important for diagnosis.

**Abbreviations & Acronyms**
ETV: extratesticular varicocele
FSH: follicle stimulating hormone
ITV: intratesticular varicocele
US: ultrasonography

**Keynote message**

In infertile males who underwent orchiopexy for undescended testes, testicular US should be performed aggressively, and if ITV is found, microsurgical varicocelectomy is considered effective in many cases and should be recommended.

## Introduction

Extratesticular varicocele (ETV) is a common condition that occurs in approximately 15% of males, most often on the left side. It is a dilation of the internal spermatic veins due to backflow toward the testes. Surgical option for varicocele repair is mainly considered when the patients complain of impaired sperm quality or chronic scrotal pain.

Apart from ETV, intratesticular varicocele (ITV) is a relatively rare condition with dilation of the intratesticular vein, which was first reported in 1992 by Weiss et al.^1^ It was reported to occur in approximately 8.6% of patients who underwent orchiopexy for undescended testis in early childhood.^2^ It is usually identified as a round‐ or oval‐shaped lesion on scrotal ultrasonography (US). To date, several studies have reported on ITV cases and the epidemiology of ITV,^3^, ^4^, ^5^, ^6^, ^7^, ^8^ but only few have reported on the surgical outcome of repair for ITV.^6^

In this case series, we report six cases of patients with a chief complaint of infertility who underwent microsurgical varicocelectomy for ITV after orchiopexy for undescended testes in their childhood.

## Case presentation

**Fig. 1:**
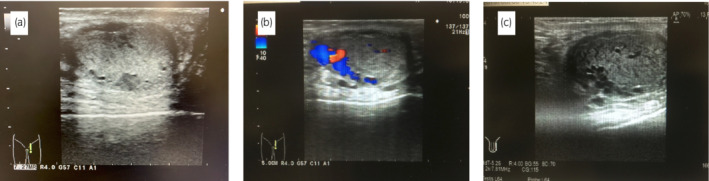
(a) Presurgical gray‐scale image of ultrasonography. Multiple hypoechoic lesions were seen within the testis. (b) Presurgical doppler image. Turbulent blood flow on Valsalva maneuver was seen in the testis. (c) Postsurgical doppler image. The blood flow has disappeared even on Valsalva maneuver.

We examined six patients with ITV who visited the male fertility outpatient clinic of the Reproduction Center of Yokohama City University Medical Center between October 2016 and July 2018. ITV was diagnosed by scrotal US when a round‐ or oval‐shaped hypoechoic lesion was identified in B‐mode, and blood congestion was identified in the color Doppler mode (Fig. 1). We usually proposed microsurgical varicocelectomy as a treatment option for ETV grade 2 or 3, and/or when ITV was identified.

They all underwent microsurgical varicocelectomy. The procedure was as follows: A 2‐ to 3‐cm transverse incision was made right above the left external inguinal ring. When the operation scar from the past orchiopexy was obvious in the inguinal area, we made the incision approximately 1 cm below the scar to avoid adhesions. After grasping the spermatic cord with Babcock forceps, we incised the external and internal spermatic fascia, and ligated the spermatic vein under microscopy. We postoperatively checked whether ETV and ITV had disappeared. Three months after surgery, they underwent semen analyses. Semen samples were provided by masturbation at our hospital with 1‐ to 5‐day intervals of sexual abstinence.

The patients' characteristics are shown in Table 1. The median age was 34 years (range: 29–46 years). The median age at which orchiopexy was performed was 4.5 years (range: 1–9 years). Five patients underwent orchiopexy on the left side, and one bilateral side. The preoperative location of the ectopic testis was unknown from the medical records. The median left testicular volume was 15 ml (range: 12–20 ml) with no evidence of obstruction in the vas deferens and epididymis; serum total testosterone level, 5.1 ng/ml; and serum FSH level, 8.3 mIU/ml. Genetic tests were not performed. The median sperm concentration and motility at the first visit were 2.8 million/ml (range, 0.4–13.5 million/ml) and 25.0% (range, 8.8%–50.0%), respectively. In all the cases, ITV and ETV occurred concomitantly (grade 2, 4; grade 1, 1; and grade 3, 1). All the patients underwent a microsurgical subinguinal varicocelectomy.

**Table 1:** Patients characteristics

No.	Age	Orchiopexy age	Partner's age	Testicular volume (mL)		Laterality of orchiopexed testis	Ipsilateral ETV grade	Endrocrinological test		Presurgical semen analysis			Number of ligated veins	Postsurgical semen analysis			Pregnancy	Delivery
				Right	Left			TT (ng/mL)	FSH (mIU/mL)	volume (mL)	sperm density (million/mL)	motility (%)		volume (mL)	sperm density (million/mL)	motility (%)		
1	46	2	40	18	12	L	2	3.0	10.1	6.8	4.4	8.8	13	3.9	8.4	10.1	−	−
2	36	5	36	20	20	B	2	2.5	8.3	1.2	1.2	9.1	27	1.0	8.3	11.0	+	+
3	32	4	35	15	12	L	2	5.6	14.3	1.5	1.0	9.0	15	1.6	11.0	27.0	+	+
4	30	1	36	16	14	L	2	5.9	2.2	3.0	9.0	44.0	20	3.2	8.1	65.4	+	+
5	29	8	30	18	20	L	3	6.2	4.4	3.0	13.5	29.0	20	1.8	23.6	15.5	−	−
6	40	9	34	18	16	L	1	4.6	8.3	2.6	0.4	50.0	30	2.1	8.2	2.4	−	−

B, bilateral sides; ETV, extratesticular varicocele; FSH, follicle stimulating hormone; L, left side; TT, total testosterone.

At 3 months after operation, the blood congestion of ITV and ETV disappeared in all the cases as confirmed on Doppler US (Fig. 1). The median postoperative sperm density and motility were 8.4 million/ml (range, 8.1–23.6 million/ml) and 21.9% (range, 2.4–65.4%), respectively. Sperm density and motility increased in five (83.3%) and four patients (66.7%), respectively. Pregnancies by the assisted reproductive techniques and live births were eventually confirmed in three couples (50.0%).

## Discussion

ITV has been reported to have a prevalence of 0.4%–2.0% among patients who underwent scrotal screening by US.^3^, ^4^, ^5^ Moreover, a higher ITV prevalence rate was reported in long‐term follow‐up study of patients who underwent prepubertal orchiopexy for undescended testes,^2^ and most of the cases occurred on the left‐side unilaterally (67.6%).^2^ Undescended testes itself could be a primary factor of spermatogenic disorders.^9^

The main reason is that the abnormal environment of the testes and exposure to elevated temperatures strongly affect germ cell differentiation.^10^ Therefore, orchiopexy between the ages of 6 and 18 months was recommended.^9^ In this study, however, the median age at which orchiopexy was performed was as late as 4.5 years.

On the other hand, ITV occurring after orchiopexy is a rare cause of male infertility and can be diagnosed only by scrotal US. To date, several studies have been conducted on ITV prevalence,^3^, ^4^, ^5^, ^6^ but few studies have investigated surgical interventions for infertility.^6^

The primary cause of ITV formation after orchiopexy has been thought to be disorder of the valvular function of the testicular veins by peeling the spermatic cord in the surgical procedure.^2^ Thereby, it is accompanied by ETV on the left side, with the same mechanism as that of ETV development, in which the left testicular vein is longer and merges perpendicularly to the left renal vein. This results in reduced venous return and blood reflux in the left testicular vein.

According to our case series, all six patients had left‐side ETV (grade 1, 1; grade 2, 4; and grade 3, 1 patients), and their sperm densities were all below the normal limit of the WHO criteria. Therefore, we proposed the surgical option, and all the patients agreed with it. Postoperatively, ITV and ipsilateral ETV disappeared in all the cases, and semen analysis revealed increase in density and motility in many patients.

This result implies that not only manipulation, but also scrotal US is essential for the screening for male infertility and that direct damage of testicular tissue due to increased temperature caused by blood congestion in ITV was considered reversible by surgery. In infertile males who underwent orchiopexy for undescended testes, testicular US should be performed aggressively, and if ITV is found, microsurgical varicocelectomy is considered effective in many cases and should be recommended. In surgery, the incision should be designed to avoid adhesions from past orchiopexy.

This case series has several limitations. Owing to the small number of cases, we did not make statistical inferences and our analyses were limited to descriptive statistics. To determine the true efficacy of surgery, a parallel‐group (surgery vs. no treatment) comparison study with a sufficient sample size is required. Another limitation is that whether the cause of spermatogenic disorder is ITV, ETV, or both is difficult to determine because ETV was accompanied with ITV in all the cases. As ITV is thought to contribute more directly to elevated testicular temperature than ETV, varicocelectomy should also be recommended for patients with ITV and without ETV.

## Author contributions

Teppei Takeshima: Conceptualization; data curation; formal analysis; writing – original draft. Kimitsugu Usui: Investigation; supervision. Shinnosuke Kuroda: Data curation; investigation; supervision. Takashi Kawahara: Supervision. Mitsuru Komeya: Supervision. Jun‐ichi Teranishi: Methodology; supervision. Hiroji Uemura: Supervision. Yasushi Yumura: Conceptualization; supervision.

## Conflict of interest

The authors declare that they have no conflicts of interest to disclose.

## Approval of the research protocol by an institutional reviewer board and the approval number

The protocol for this case series has been approved by a suitably constituted Ethics Committee of the institution (Committee of Yokohama City University Medical Center, Approval No. B2103000444).

## Informed consent

All patients provided written informed consent prior to their participation.

## Registry and the registration no. of the study/trial

N/A.
